# Tumor-promoting properties of karyopherin β1 in melanoma by stabilizing Ras-GTPase-activating protein SH3 domain-binding protein 1

**DOI:** 10.1038/s41417-022-00508-8

**Published:** 2022-07-28

**Authors:** Fan Yang, Lin Li, Zhenzhen Mu, Pengyue Liu, Ying Wang, Yue Zhang, Xiuping Han

**Affiliations:** Department of Dermatology, Shengjing Hospital of China Medical University, Shenyang, 110004 Liaoning PR China

**Keywords:** Tumour biomarkers, Biomarkers

## Abstract

The nuclear import receptor karyopherin β1 (KPNB1), a member of the Karyopherin protein family, is reported to be overexpressed in various cancers and promote carcinogenesis. By analyzing the correlation between the expression of KPNB1 and the overall survival rate of melanoma patients, we found that melanoma patients with higher expression of KPNB1 had worse survival. Furthermore, the database analyzed that the KPNB1 mRNA level was higher in melanoma samples than that in skin nevus tissues. We thus proposed that KPNB1 played a role in promoting melanoma development, and conducted gain-of- and loss-of-function experiments to test our hypothesis. We found that KPNB1 knockdown significantly retarded the growth and metastasis of melanoma cells in vitro and in vivo, and increased their sensitivity towards the anti-tumor drug cisplatin. KPNB1 overexpression had opposite effects. Notably, in melanoma cells, KPNB1 overexpression significantly decreased Ras-GTPase-activating protein SH3 domain-binding protein 1 (G3BP1) protein level, which was also overexpressed in melanoma samples and enhanced malignant behaviors of melanoma cells. We further demonstrated that KPNB1 overexpression induced deubiquitination of G3BP1, and prevented its degradation. However, KPNB1 overexpression hardly affected the nuclear translocation of G3BP1. Additionally, alterations induced by KPNB1 overexpression were partly reversed by G3BP1 inhibition. Therefore, the results suggest that KPNB1 may promote melanoma progression by stabilizing the G3BP1 protein. KPNB1-G3BP1 axis represents a potential therapeutic targetable node for melanoma.

## Introduction

Melanoma is a deadly skin cancer derived from melanocytes [1]. The incidence of malignant melanoma is on the rise worldwide and melanoma treatment has become an important socio-economic problem. Most melanomas occur in pre-existing moles in areas exposed to the sun and spread rapidly through the vascular system if not detected and removed early [2]. Surgical excision of the primary tumor and immunotherapies improve the survival rate of cancer patients [3, 4]. However, metastasis and chemotherapy resistance make cancer treatment not optimistic [5, 6]. Therefore, exploring molecular markers in melanoma progression for predicting the course of the disease and providing new targets for therapy are of great significance.

Karyopherin β1 (KPNB1) is a major nuclear importer of the karyopherin family and has been reported to regulate diverse cellular process, such as apoptosis [7], proliferation [8], and invasion [9]. Many researchers have demonstrated the important role of KPNB1 in cancer development. Currently, KPNB1 has been proved to play a promoting role in many types of cancer, such as non-small cell lung [8], prostate cancer [10], and colorectal cancer [11]. Talantov et al. [12] analyzed the differentially expressed genes in 45 primary melanoma and 18 nevus tissue specimens on an Affymetrix Hu133A microarray and found that KPNB1 was highly expressed in melanoma samples [12]. However, little is known about the function and underlying regulatory mechanism of KPNB1 in melanoma.

Ras-GTPase-activating protein SH3 domain-binding protein 1 (G3BP1) is an SH3 domain-binding protein that has been reported to play a pro-cancer role in many types of cancer, including prostate cancer [13], breast cancer [14], and renal cell carcinoma [15]. Functional studies proved that G3BP1 promoted the malignant behaviors of cancer cells, including cell survival, metastasis, and the chemotherapy resistance of cancer cells [16, 17]. Furthermore, G3BP1 has been reported to mediate the promoting effect of circ_0119872 on uveal melanoma progression [18]. Those findings indicate that G3BP1 may be a promising target for cancer therapy, including melanoma. However, Mao et al. [19] revealed that the cytosolic P53RRA-G3BP1 interaction displaced p53 from a G3BP1 complex, resulting in greater p53 retention in the nucleus, which led to cell-cycle arrest, apoptosis, and ferroptosis, indicating that G3BP1 may also function as a tumor suppressor. The results of the Cancer Genome Atlas (TCGA) database analyzed by the Gene Expression Profiling Interactive Analysis (GEPIA) website showed that G3BP1 was overexpressed in melanoma samples. However, the function of G3BP1 in melanoma progression remains unclear.

It has been previously reported that KPNB1 inhibition caused impaired cellular proteostasis and increased ubiquitination of both p65 and total proteins in glioblastoma cells [20], which suggested that KPNB1 probably had functions similar to the deubiquitination enzyme. HitPredict software (http://www.hitpredict.org/) analyzed that there was binding between KPNB1 and G3BP1 at the protein level. We thus speculated that KPNB1 might affect G3BP1 expression through deubiquitination. Altogether, we mainly investigated the functions of KPNB1 and G3BP1 in melanoma and their regulatory relationship.

## Materials and methods

### Cell culture

Human melanoma cell lines A375 and A2058 were maintained in Dulbecco’s Modified Eagle’s medium (DMEM, Servicebio, Wuhan, China) and A875, SK-MEL-1, and SK-MEL-2 were cultured in Minimum Essential Medium (MEM, Solarbio, Shanghai, China). All mediums were supplemented with 10% fetal bovine serum (FBS, Tianhang Biotech, Zhejiang, China) and cultured at a 37 °C incubator with 5% CO_2_. Melanoma cells were purchased from Procell (Wuhan, China) or iCell Bioscience (Shanghai, China). Primary human skin melanocytes cells were purchased from iCell Bioscience (Shanghai, China) and cultured with special medium (iCell Bioscience).

### Lentiviral vector construction and infection

The specific short hairpin RNAs (shRNAs) targeting KPNB1 were inserted into the pLVX-shRNA1 lentivirus (LV) vector (Fenghui Biotech, Changsha, China) to silence KPNB1 expression. The sequences of shRNA were 5’-cccTTCTCCGAACGTGTCACGTttcaagagaACGTGACACGTTCGGAGAAttttt-5’ (sh-NC); 5’-cccCAGAGCAGCTACAAGATAAATttcaagagaATTTATCTTGTAGCTGCTCTGttttt-3’ (sh1- KPNB1); 5’-cccGCTCAAACCACTAGTTATACAttcaagagaTGTATAACTAGTGGTTTGAGCttttt-3’ (sh2-KPNB1). To upregulate KPNB1 expression, a pLVX-IRES-puro lentivirus vector (Fenghui Biotech) containing a coding sequence of KPNB1 was constructed. The shRNA lentivirus was infected cells at a multiplicity of infection of 150 and the viral titer for the lentivirus vector was 10^8^ TU/ml. After 48 or 72 h infection, cell lines with stable low or high expression of KPNB1 were obtained by the inclusion of antibiotics in the culture medium.

### Cells treatment and transfections

For follow-up experiments, cells stably overexpressing or silencing KPNB1 were treated with cisplatin (DDP; 0, 4, 8, 16, 32, or 64 μM; Meilunbio, Dalian, China) for 48 h, treated with cycloheximide (CHX; 12.5 µg/mL; Meilunbio) for 0, 2, 4 or 8 h, or treated with MG132 (10 μM; MedChemExpress, Shanghai, China) for 6 h. Small interfering RNA (siRNA) of G3BP1 was purchased from JST Scientific (Wuhan, China). The sequences were: 5’-GCCACACCAAGAUUCGCCATT-3’ (si-NC); 5’-UGGCGAAUCUUGGUGUGGCTT-3’ (si-G3BP1). The si-NC or si-G3BP1 was transfected into melanoma cells with KPNB1 knockdown/overexpression for 48 h and the transfections were mediated by Lipofectamine 3000 (Invitrogen, Carlsbad, CA, USA).

### Real-time quantitative PCR (RT-qPCR)

Quantitative RT-PCR was performed to detect the mRNA level of genes. Total RNA was extracted from cells using TriPure reagent, chloroform, and isopropanol following the manufacturer’s instructions. For mRNA detection, RNA was reverse transcribed by using BeyoRT II M-MLV reverse transcriptase (Beyotime, Shanghai, China), and quantitative RT-PCRs were conducted using gene-specific primers, SYBR Green (Solarbio), and 2 × Taq PCR Master Mix. Quantitative normalization was performed using the expression of β-actin. Relative mRNA expression was calculated using the 2^−ΔΔCT^ method. Primer sequences were provided in Supplementary Table 1.

### Western blot

Total protein was obtained from cells and tissues by using RIPA buffer (Beyotime) and phenylmethanesulfonyl fluoride (Beyotime). Nuclear protein was extracted from cells by using a Nuclear Protein Extraction kit (Beyotime) according to the manufacturer’s protocol. Cell lysates (15–30 μg protein) were loaded per lane and separated on SDS-PAGE gels (8%, 10%, or 15% separation gel). Next, the proteins were blotted onto the PVDF membrane. After being treated with 5% bovine serum albumin for 1 h, the membrane was incubated with primary antibodies (Shown in Supplementary Table 2) overnight at 4 °C followed by the incubation of the secondary antibody for 40 min. The secondary antibodies used were as follows: goat anti-rabbit IgG (No. SA00001-2; Proteintech; 1:10000) and goat anti-mouse IgG (No. SA00001-1; Proteintech; 1:10000). After washing with TBST, immunoreactions were visualized by using an ECL reagent solution (7 Sea biotech, Shanghai, China). Quantitative normalization was performed using the expression of β-actin. Histone H3 served as an internal control for nuclear protein detection.

### Cell counting kit-8 (CCK-8) assay

The 5 × 10^3^ melanoma cells were seeded into a 96-well plate. At 0, 24, 48, 72, and 96 h or treated with 0, 4, 8, 16, 32, and 64 μM DDP for 72 h, 10 μL CCK-8 (KeyGEN, Jiangsu, China) was added to each well and incubated for 2 h in a 37 °C incubator with 5% CO_2_. The absorbance at 450 nm was measured by a microplate reader (Biotek, Winooski, VT, USA).

### Plate colony formation assay

Cells were seeded in Petri dishes (600 cells per petri dish) and then placed in a 37 °C incubator with 5% CO_2_ for 2 weeks. After washing with PBS, cells were fixed with 4% paraformaldehyde for 20 min at room temperature and stained with Wright-Giemsa stain solution (KeyGEN) for 5 min. After washing with water, cell count was performed under a microscope (Olympus, Tokyo, Japan).

### Flow cytometry assay

Cells were cultured in a 37 °C incubator with 5% CO_2_ until adhere to the wall. For cell cycle detection, cells were harvested and fixed with ethanol (70%) for 2 h at 4 °C. For cytometry analysis, cells were treated with propidium iodide (PI; 25 μL) and RNase A (10 μL) for 30 min at room temperature in a dark room. Finally, cell cycle distribution was analyzed by a flow cytometer (ACEA Biosciences, San Diego, CA, USA). For apoptosis detection, cells were labeled with Annexin V-FITC (5 μL) and PI (5 μL) using an Apoptosis Detection kit (KeyGEN) according to the manufacturer’s instructions. After incubation for 15 min in the dark, apoptosis was measured by using a flow cytometer (ACEA Biosciences).

### Wound healing

After the cells were cultured to the confluent state, 1 μg/mL mitomycin C was added into the medium for 1 h. Next, the monolayer of cells was scratched with a 200 μL pipette tip, and images of the scratch at time 0 h were captured. After the cells were cultured in a serum-free medium for 24 h in a 37 °C incubator with 5% CO_2_, images of the scratch at the time 24 h were obtained.

### Transwell assay

The Transwell chamber was placed into a 24-well plate and coated with Matrigel. Next, cells (3 × 10^4^ per well) were added to the upper chamber. The culture medium (800 μL) supplemented with 10% FBS was added to the lower chamber. After 24 h of incubation, cells were fixed with 4% paraformaldehyde for 25 min and stained with 0.4% crystal violet staining solution for 5 min. Images were obtained using a microscope (Olympus) and the number of migrated cells was counted.

### In vivo tumor growth and pulmonary metastasis assay

All the mouse experiments were approved by the Ethics Committee of Shengjing Hospital of China Medical University and followed the National Institutes of Health Guide for the care and use of laboratory animals.

BALB/c nude mice aged 6 weeks were purchased from HFK Biotechnology Co., Ltd. (Beijing, China). For tumor growth, healthy nude mice were randomly divided into 5 groups (*n* = 8) named sh-NC, sh1-KPNB1, sh2-KPNB1, OV-NC, and OV-KPNB1. Cells (2 × 10^6^ cells) stably overexpressing (SK-MEL-2 cells) or silencing (A375 cells) KPNB1 were subcutaneously into nude mice. Tumor volume was detected every 3 days, 21 days later, tumor tissues were taken out and weighed. The tumor tissues were fixed with 4% paraformaldehyde or stored in liquid nitrogen.

For pulmonary metastasis detection, animal grouping is the same as above. Cells (2 × 10^6^ cells) infected with recombinant pLKO.1-EGFP-puro lentivirus (Fenghui Biotech) with KPNB1 overexpression (SK-MEL-2 cells) or silencing (A375 cells) were injected into nude mice through the tail vein. Seven weeks post-injection, tumor cells in the lung were reacted by a small animal living imager (Multimodal pro Light source 400 W; Bruker, Bremen, Germany). Next, nude mice were euthanized and tumor tissue was removed. The lung tissues were fixed with 4% paraformaldehyde for subsequent experiments. Based on previous studies, SK-MEL-2 and A375 cells were used for in vivo metastasis experiments [21, 22].

### Immunohistochemistry (IHC)

Tumor tissues fixed with 4% paraformaldehyde were embedded in paraffin and sectioned (5 μm). Slides were then deparaffinized and rehydrated with graded alcohols. After blocking with normal goat serum for 15 min at room temperature, the slides were incubated with primary antibody against PCNA (No. A0264; Abclonal, Wuhan, China; 1:50) overnight at 4 °C. After washing with PBS, the slides were treated with paired secondary antibody (No. #31460; Thermo Fisher Scientific, Waltham, MA, USA; 1:500) for 1 h at 37 °C. The slides were then incubated with DAB color development reagent (Solarbio) and counterstained with hematoxylin. After dehydration and sealing, the slides were observed and pictured using a microscope (400×, Olympus).

### Hematoxylin and eosin (H&E) staining

Lung tissues fixed with 4% paraformaldehyde were sectioned and stained with H&E to observe the lung metastasis of melanoma cells. Images were captured and pulmonary nodules were counted by using a microscope (40×, Olympus). The number of pulmonary nodules in the same area was quantitatively analyzed.

### Immunofluorescence (IF) staining

Melanoma cells were fixed with paraformaldehyde (4%) for 15 min. After washing with PBS three times, cells were treated with 0.1% Triton X-100 for 30 min. Then, cells were incubated with normal goat serum (Solarbio) for 15 min at room temperature, followed by the incubation of primary antibody (G3BP1; No. 13057-2-AP; Proteintech; 1:50 or/and KPNB1; No. MAB8209-SP; Novus; 1:50) at 4 °C overnight. Cells were then incubated with goat anti-rabbit IgG labeled with Cy3 (No. A0516; Beyotime; 1:200) or Goat anti-mouse IgG labeled with FITC (NO. SA00003-1; Proteintech; 1:100) for 60 min in the dark at room temperature. After cells were washed in PBS and mounted with DAPI (No. D106471-5mg; Aladdin, Shanghai, China), a fluorescence microscope was used to obtain the images (400×, Olympus). Pearson correlation coefficient was analyzed based on previous studies [23].

### Co-immunoprecipitation (co-IP) assay and ubiquitination assay

Proteins were isolated from melanoma cells and melanoma cells with KPNB1 knockdown or overexpression using Western and IP Lysis Solution (Beyotime). Next, the proteins were treated with IP-indicated antibody (1 μg) or paired mouse/rabbit-IgG (1 μg, Beyotime and Proteintech) at 4 °C overnight. Extracted proteins served as input controls. Protein A agarose beads were used to capture antigen-antibody complexes. After washing with PBS, the complexes were resuspended in loading buffer and boiled for 5 min to free the antigen and antibody. After, centrifugation, the supernatants were collected for western blot analysis. Primary antibodies used for co-IP were shown in Supplementary Table 3.

### Statistical analysis

Statistical analysis was conducted by using GraphPad Prism 8.0 software. One-way ANOVA combined with Tukey’s post hoc test and unpaired *t*-test was used to analyze significance among groups. It is acceptable for small sample sizes when the effect size > 0.8 [24–26]. Effect sizes were calculated by Post Hoc Power Analysis to confirm the power of the analysis [27, 28]. All data were shown as the mean ± standard deviation (SD). *P* < 0.05 indicated statistical significance.

## Results

### Increased KPNB1 levels are observed in melanoma tissues and cell lines and indicate a poor prognosis

**Fig. 1:**
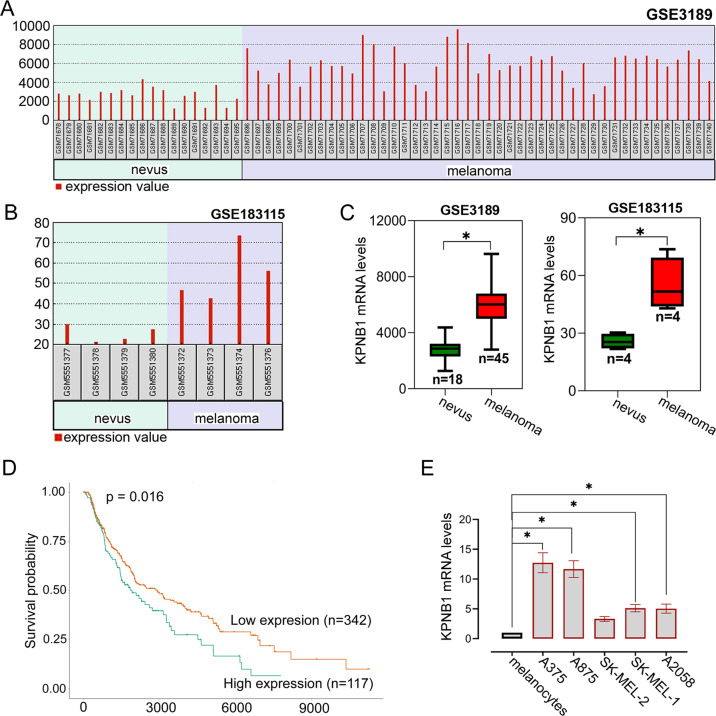
Increased KPNB1 levels are observed in melanoma tissues and cell lines and indicate a poor prognosis. **A**–**C** Data about KPNB1 mRNA expression in primary melanoma and benign skin nevi specimens were obtained from GEO DataSets GSE3189 and GSE183115 and analyzed. **D** Survival analysis of melanoma patients with KPNB1 overexpression or silencing was conducted using UALCAN website. **E** KPNB1 mRNA expression was measured in melanocytes and melanoma cell lines (A2058, SK-MEL-1, SK-MEL-2, A875, and 375). *N* = 3. Data were shown as the mean ± SD.

We analyzed the microarray analysis data on KPNB1 mRNA expression in 45 primary melanomas and 18 benign skin nevus tissues in the previous study [12]. The results showed that KPNB1 expression was clearly higher in melanomas tissues (*n* = 45) than that in benign skin nevus tissues (*n* = 18) (Fig. 1A, C). Moreover, results of the GSE183115 database also confirmed that KPNB1 mRNA expression was significantly increased in melanomas tissues (*n* = 4) compared with nevus tissues (*n* = 4) (Fig. 1B, C). UALCAN (an interactive web-portal to perform in-depth analyses of TCGA gene expression data) analyzed that higher KPNB1 expression indicated poorer prognosis **(**Fig. 1D**)**. We further measured KPNB1 mRNA expression in melanoma cell lines and found that KPNB1 was highly expressed in melanoma cell lines (A2058, SK-MEL-1, SK-MEL-2, A875, and A375) compared with melanocytes (Fig. 1E). Overall, the results suggested that KPNB1 expression was upregulated in melanoma and was associated with a poor prognosis.

### KPNB1 promotes melanoma cell growth, invasion, and migration in vitro

**Fig. 2:**
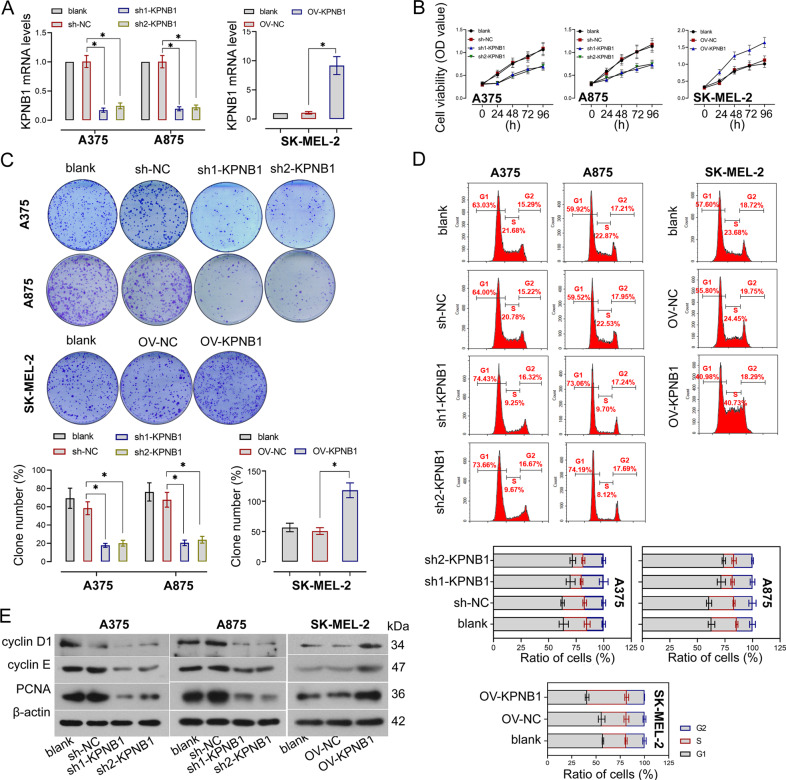
KPNB1 promoted cell proliferation in melanoma cell lines. **A** The mRNA expression of KPNB1 in KPNB1-overexpressed or -silenced cells were measured by real-time quantitative PCR. **B** Cell viability was determined by the CCK-8 assay. **C** Representative colony formation assay of melanoma cells with KPNB1 overexpression or knockdown. **D** Cell cycle distribution was measured by flow cytometry. **E** Relative protein levels of cyclin D1, cyclin E, and PCNA were detected by Western blot. *N* = 3. Data were shown as the mean ± SD. *P < 0.05 was determined by One-way ANOVA combined with Tukey’s post hoc test.

**Fig. 3:**
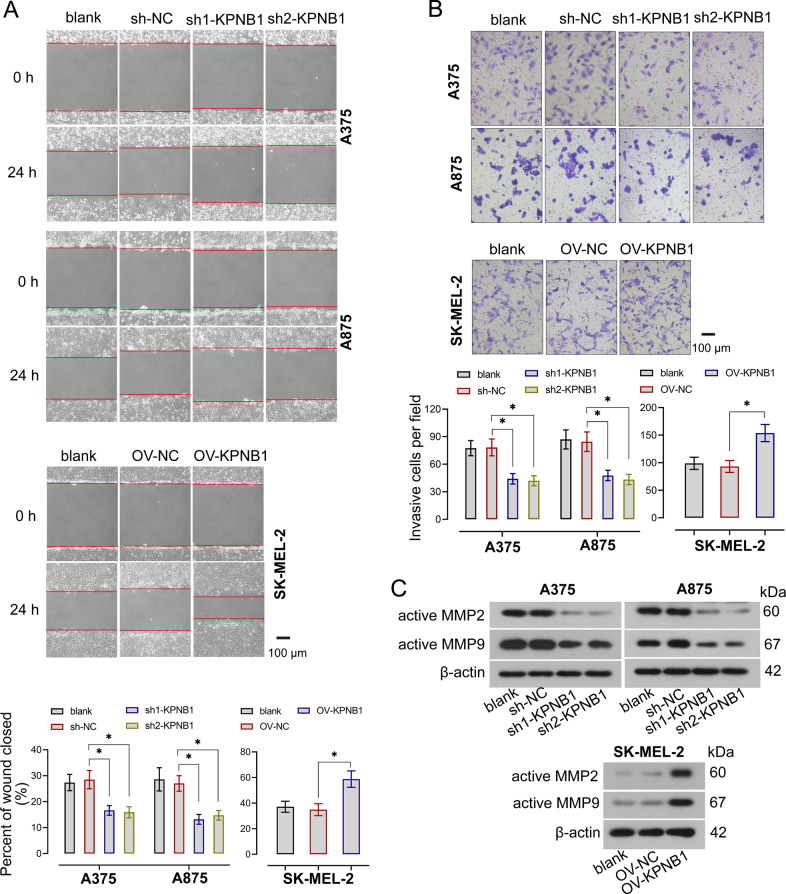
KPNB1 promoted invasion and migration in melanoma cell lines. **A** Wound healing rate of melanoma cells with KPNB1 overexpression or silencing. Scale Bar = 100 μm. **B** Invasive cell numbers of melanoma cells with KPNB1 overexpression or silencing. Scale Bar = 100 μm. **C** The active MMP2 and MMP9 protein expression was examined by Western blot. *N* = 3. Data were shown as the mean ± SD. *P < 0.05 was determined by One-way ANOVA combined with Tukey’s post hoc test.

To evaluate the effect of KPNB1 on the oncogenic cellular behavior of melanoma cells, KPNB1 expression was silenced or upregulated in melanoma cells. The knockdown and overexpression efficiencies of KPNB1 were verified by RT-qPCR (Fig. 2A). CCK-8 analysis showed that cell proliferation was significantly decreased in KPNB1-silenced cells but increased in cells with KPNB1 overexpression (Fig. 2B). Colony numbers were obviously lower in KPNB1-silenced cells and higher in KPNB1-overexpressing cells than that in the corresponding NC cells (Fig. 2C). Furthermore, cell cycle distribution was examined by flow cytometry and observed that KPNB1 downregulation arrested more cells in the G1 phase and decreased the cells in the G2 phase but KPNB1 overexpression had the opposite effect (Fig. 2D). Meanwhile, cell proliferation was also confirmed by the detection of proliferation-related protein expression, in which cyclin D1, cyclin E, and PCNA protein expression was significantly downregulated by KPNB1 silencing but upregulated by KPNB1 overexpression (Fig. 2E, Supplementary Fig. 3A). Furthermore, wound healing assay and Transwell assay analyzed that wound closure was delayed and cell invasion was suppressed in cells with KPNB1 knockdown, whereas KPNB1 upregulation promoted wound closure and cell invasion (Fig. 3A, B, Supplementary Fig. 1C). Moreover, changes in migration and invasion markers were measured by Western blot and indicated that KPNB1 inhibition decreased MMP2 and MMP9 protein levels, whereas their levels were increased by KPNB1 elevation (Fig. 3C, Supplementary Fig. 3B). Overall, those results indicated that KPNB1 contributed to the growth, migration, and invasion capabilities of melanoma cells.

### KPNB1 promotes melanoma cell growth and metastasis in vivo

**Fig. 4:**
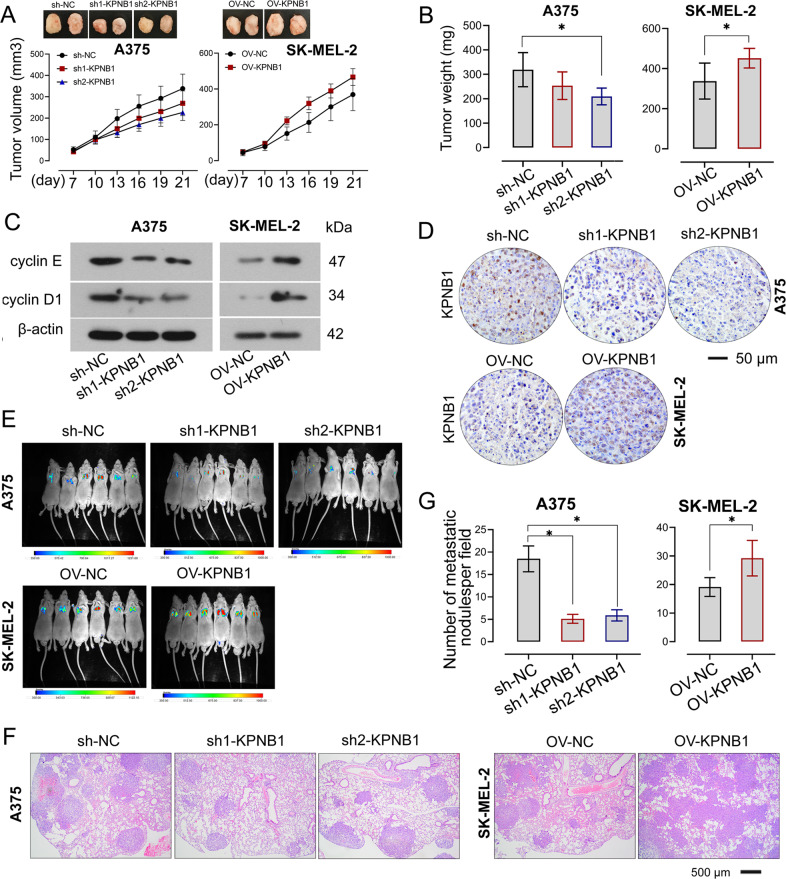
KPNB1 promotes melanoma cell growth and metastasis in vivo. **A, B** The tumor volume and weight of mice were measured after KPNB1-overexpressed or silenced cells injection. **C** Relative protein expression of cyclin E and cyclin D1 in tumor tissues was measured by Western blot. **D** KPNB1 expression in tumor tissues was measured by immunohistochemistry. Scale Bar = 50 μm. **E** Melanoma cells with KPNB1 overexpression or silencing were injected via the tail vein into mice and the bioluminescence images were taken 7 weeks after injection. **F** H&E staining was used to detect lung metastases of melanoma cells. Scale Bar = 500 μm. **G** Number of lung nodules in H&E-stained lung sections in each group. *N* = 8. Data were shown as the mean ± SD. *P < 0.05 was determined by unpaired *t*-test and One-way ANOVA combined with Tukey’s post hoc test.

To validate the in vitro results on the role of KPNB1 in melanoma cell growth and metastasis, the in vivo effects of KPNB1 silencing/overexpression on the tumor xenograft growth and pulmonary metastasis in nude mice were investigated. It was shown that subcutaneous injection of melanoma cells with KPNB1 silencing significantly reduced tumor volume and weight, whilst KPNB1-overexpressed cells injection increased the tumor volume and weight of mice (Fig. 4A, B). Meanwhile, KPNB1 expression in tumor tissues was upregulated by KPNB1-overexpressed cells injection, whereas KPNB1-silenced cells injection significantly downregulated its expression (Fig. 4D). Furthermore, relative protein levels of cyclin E and cyclin D1 in tumor tissues were inhibited by KPNB1 silencing but enhanced by KPNB1 overexpression (Fig. 4C, Supplementary Fig. 3C). Following tail vein injection of melanoma cells with KPNB1 knockdown or overexpression in nude mice, lung metastasis was detected 7 weeks post-injection. The results showed that KPNB1-overexpressed cells injection displayed lung colonization and increased lung nodule, whereas pulmonary metastases and lung nodule were reduced in mice injected with KPNB1-silenced cells (Fig. 4E–G). Taken together, those findings suggested that KPNB1 promoted the growth and metastatic capacity of melanoma cells in vivo.

### KPNB1 decreases the DDP sensitivity of melanoma cells

**Fig. 5:**
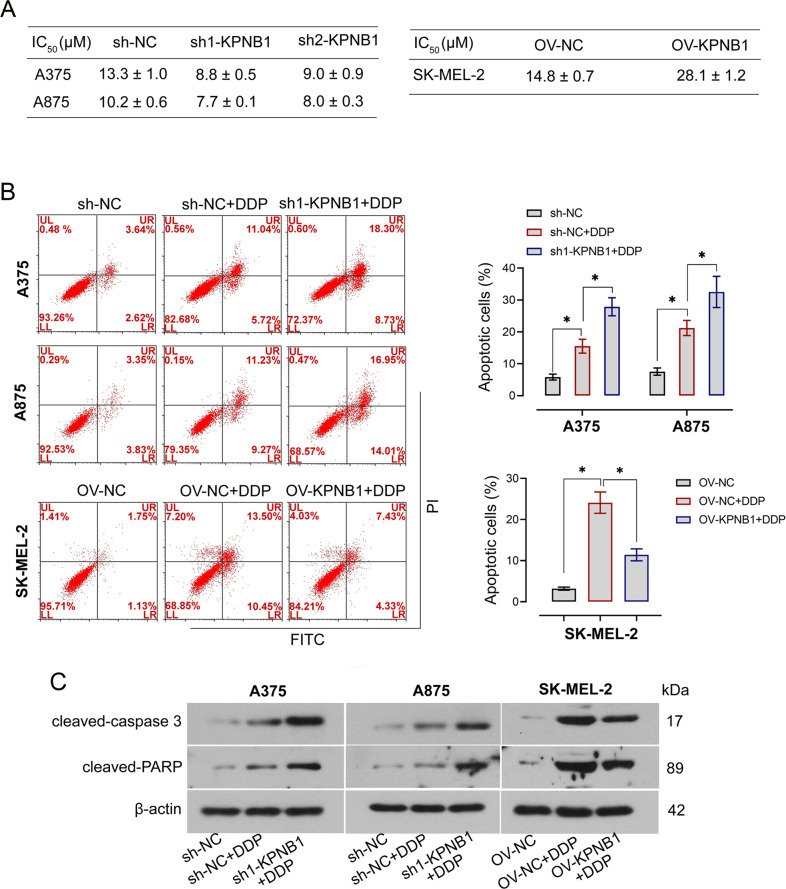
KPNB1 decreases DDP sensitivity of melanoma cells. **A** After the KPNB1-silenced or overexpressed cells were treated with DDP (0, 4, 8, 16, 32, or 64 μM), the IC_50_ value was calculated. **B** The apoptosis of KPNB1-silenced or -overexpressed cells treated with DDP. **C** Relative cleaved caspase 3 and cleaved PARP protein expression in KPNB1-silenced or overexpressed cells treated with DDP. *N* = 3. Data were shown as the mean ± SD. *P < 0.05 was determined by One-way ANOVA combined with Tukey’s post hoc test.

To explore the effects of KPNB1 expression on the DDP sensitivity of melanoma cells, melanoma cells in each group were treated with 0, 4, 8, 16, 32, or 64 μM DDP, respectively, and then the half-maximal inhibitory concentration (IC_50_) value was calculated. It was shown that compared with their NC cells, the IC_50_ value was lower in KPNB1-silenced cells and higher in KPNB1-overexpressed cells (Fig. 5A). Furthermore, we also explored the effects of KPNB1 on DDP-mediated apoptosis. Results of flow cytometry analysis showed that DDP treatment caused a significant increase in apoptotic rate, and melanoma cells with lower KPNB1 expression showed more apoptosis upon DDP exposure (Fig. 5B). Conversely, KPNB1 overexpression protected melanoma cells from DDP-mediated apoptosis (Fig. 5B). Furthermore, apoptosis-related factors were measured by Western blot to further verify the effects of KPNB1 on DDP-mediated apoptosis (Fig. 5C). The results showed that KPNB1 knockdown further increased DDP-induced cleaved caspase 3 and cleaved PARP expression but KPNB1 overexpression showed the opposite effect (Fig. 5C, Supplementary Fig. 3D). Taken together, the results above indicated that KPNB1 might antagonize the cellular sensitivity towards DDP.

### KPNB1 enhances the stability of G3BP1

**Fig. 6:**
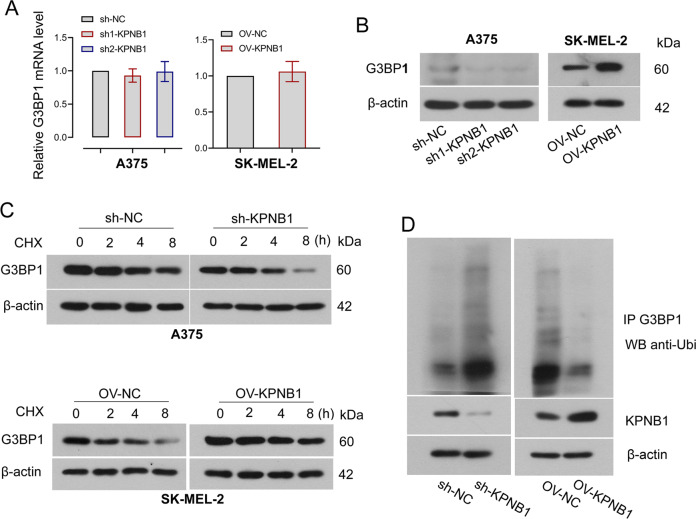
KPNB1 enhances the stability of G3BP1 in melanoma cells. **A** Relative mRNA expression of G3BP1 in melanoma cells was measured by Real-Time quantitative PCR. **B** G3BP1 protein levels in cells with KPNB1 overexpression or silencing were examined by Western blot. **C** G3BP1 protein expression was measured in KPNB1-silenced or overexpressed cells treated with 12.5 µg/mL cycloheximide (CHX) for 0, 2, 4, or 8 h. **D** Co-immunoprecipitation assay combined with ubiquitination assay were used to detect the ubiquitination of G3BP1 in KPNB1-silenced or overexpressed cells. *N* = 3. Data were shown as the mean ± SD. *P < 0.05 was determined by unpaired *t*-test and One-way ANOVA combined with Tukey’s post hoc test.

Considering that KPNB1 is related to protein proteostasis and unfolded protein response [20]. We further explored the mechanism by which KPNB1 is involved in melanoma progression. We first analyzed the correlation between KPNB1 and G3BP1 at the transcriptional level by using the GEPIA website (http://gepia.cancer-pku.cn/). The results showed that KPNB1 and G3BP1 were positively correlated at the RNA level (Supplementary Fig. 2). However, our results showed that KPNB1 knockdown or overexpression had no significant effect on the G3BP1 mRNA level (Fig. 6A), indicating that the correlation between KPNB1 and G3BP1 may not prove the regulatory relationship between them. However, decreased protein levels in KPNB1-silenced cells and increased protein levels in KPNB1-overexpressed cells were observed (Fig. 6B, Supplementary Fig. 3E), which indicated that KPNB1 might affect the expression of G3BP1 at the protein level. Moreover, results of co-IP and immunofluorescence double staining assays displayed that KPNB1 and G3BP1 were co-localized and interacted at the protein level (Supplementary Fig. 1A, B). Furthermore, the effect of KPNB1 on the stability of G3BP1 protein was further studied by treating KPNB1 overexpressing or silencing cells with CHX (an inhibitor of protein synthesis). As indicated in Fig. 6C that CHX treatment decreased KPNB1 and G3BP1 protein expression, which was further enhanced by KPNB1 silencing but rescued by KPNB1 overexpression, suggesting that KPNB1 might increase the stability of G3BP1. Ubiquitin-mediated proteasomal degradation is considered the major factor affecting protein stability in eukaryotes [29]. Co-IP assay and ubiquitination assay analyzed that ubiquitination of G3BP1 was significantly increased in cells with KPNB1 knockdown, whereas overexpression of KPNB1 reduced its ubiquitination (Fig. 6D). Taken together, those results suggested that KPNB1 might up-regulate the expression of G3BP1 by affecting the stability of G3BP1.

### KPNB1 does not affect the nuclear transport of G3BP1

**Fig. 7:**
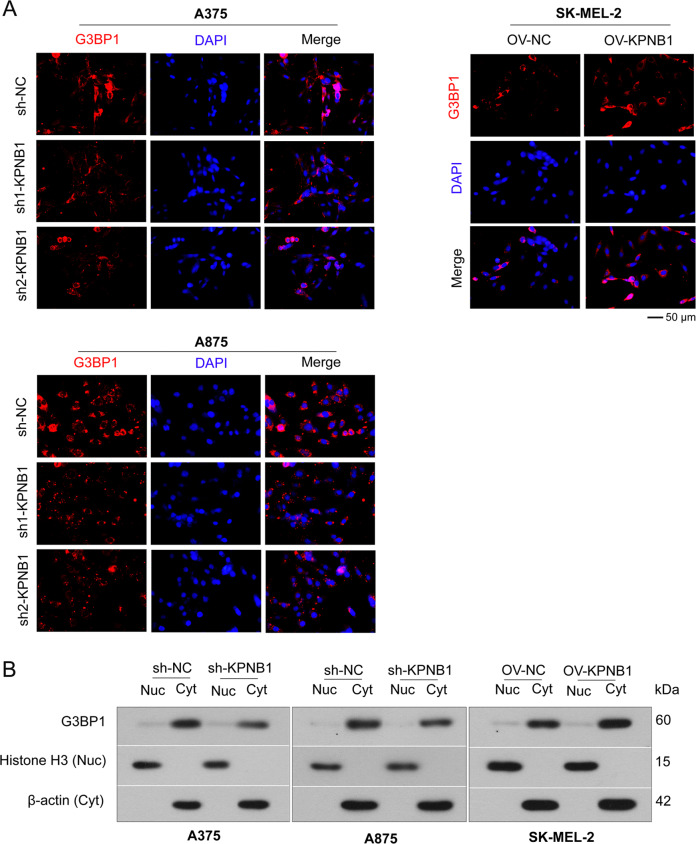
KPNB1 does not affect the nuclear transport of G3BP1. **A** The localization and expression of G3BP1 in cells with KPNB1 overexpression or silencing were detected by immunofluorescence staining. Scale Bar = 50 μm. **B** Western blot was used to analyze the protein expression of G3BP1 in the nucleus and cytoplasm in KPNB1-silenced or overexpressed cells. *N* = 3.

As an important regulator of the nuclear importer, whether KPNB1 regulates the nuclear import of G3BP1 was further investigated. The localization and expression of G3BP1 in cells were detected by IF staining, and we found that G3BP1 was mainly distributed in the cytoplasm, and KPNB1 knockdown reduced the expression of G3BP1 in the cytoplasm, whereas KPNB1 overexpression had the opposite effect (Fig. 7A). However, neither KPNB1 knockdown nor overexpression affects the nuclear translocation of G3BP1 (Fig. 7A). Consistently, Western blot analyzed that the protein expression of KPNB1 was only positively correlated with the protein expression of G3BP1 in the cytoplasm but did not regulate the expression of G3BP1 in the nucleus (Fig. 7B). The results indicated that KPNB1 did not affect the nuclear transport of G3BP1.

### G3BP1 promotes the malignant phenotype of melanoma cells

**Fig. 8:**
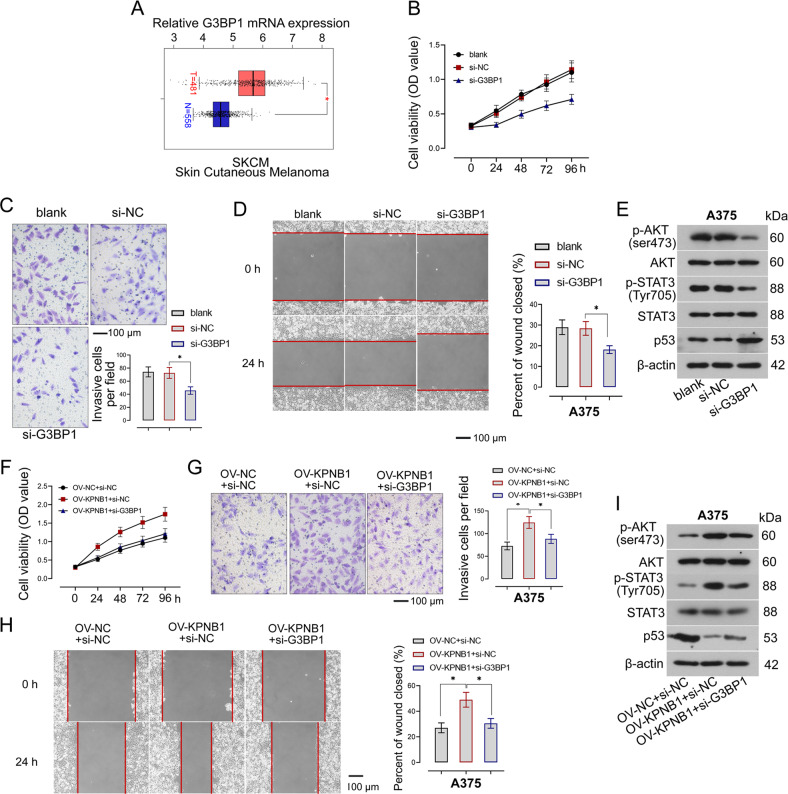
G3BP1 promotes the malignant phenotype of melanoma cells and mediates the regulation of KPNB1 on melanoma progression. **A** G3BP1 mRNA expression in melanoma samples and normal samples was analyzed by the GEPIA website. **B** Cell viability in G3BP1-silenced cells was detected by using the CCK-8 assay. **C**, **D** Cell invasion and migration in cells with G3BP1 downregulation were measured. Scale Bar = 100 μm. **E** Relative expression of p-AKT (ser473), AKT, p-STAT3 (Tyr705), STAT3, and p53 in G3BP1-silenced cells was measured by Western blot. **F**–**H** Cell viability, invasion, migration was measured in KPNB1-overexpressed cells transfected with si-G3BP1 or si-NC. Scale Bar = 100 μm. **I** The protein expression of p-AKT (ser473), AKT, p-STAT3 (Tyr705), STAT3, and p53 in KPNB1-overexpressed cells transfected with si-G3BP1 or si-NC was detected by Western blot. *N* = 3. Data were shown as the mean ± SD. *P < 0.05 was determined by One-way ANOVA combined with Tukey’s post hoc test.

GEPIA analyzed that G3BP1 was highly expressed in melanoma samples (Fig. 8A). The effects of G3BP1 on the malignant phenotypes of melanoma cells were explored by transfecting with G3BP1 siRNA. As presented in Fig. 8B, G3BP1 inhibition clearly decreased cell viability. In addition, the invasion and migration capacities of melanoma cells were also significantly decreased in G3BP1-silenced cells (Fig. 8C, D). Overall, the results demonstrated that G3BP1 promoted melanoma progression. Furthermore, we measured the markers of the main pathways regulating the malignant phenotype of cells, in which G3BP1 knockdown markedly downregulated the phosphorylation levels of AKT and signal transducer and activator of transcription 3 (STAT3) but increased p53 protein level (Fig. 8E, Supplementary Fig. 3F).

### G3BP1 mediates the regulation of KPNB1 on melanoma progression

Given that KPNB1 could upregulate G3BP1 expression, we next investigate the role of G3BP1 in KPNB1–mediated melanoma progression. Our results showed that the increase in cell viability, invasion, and migration induced by KPNB1 overexpression was restored by G3BP1 silencing (Fig. 8F–H). Moreover, KPNB1 elevation significantly increased phosphorylation levels of AKT and STAT3 but decreased p53 expression, and these phenomena were reversed by KPNB1 knockdown (Fig. 8I, Supplementary Fig. 3G). Therefore, G3BP1 might mediate the regulation of KPNB1 on melanoma progression.

## Discussions

In the present study, we demonstrated that KPNB1 was highly expressed in melanoma tissues and cells and survival was worse among melanoma patients with high KPNB1 expression. KPNB1 overexpression contributed to melanoma cell growth and metastasis in vivo and in vitro. Furthermore, KPNB1 overexpression induced deubiquitination of G3BP1, and prevented its degradation but hardly affected the nuclear translocation of G3BP1. Alterations induced by KPNB1 overexpression were partly reversed by G3BP1 inhibition. Altogether, our study elucidates the tumor-promoting function of KPNB1 through upregulating G3BP1.

The treatment of melanoma, one of the most aggressive human malignancies, has been zrevolutionized by the advent of novel targeted and immunotherapies. Although the diagnosis of cutaneous malignant melanoma is usually based on histopathological criteria, these criteria may sometimes be insufficient to distinguish melanoma from certain types of benign moles. Tumor resection is the mainstay of cancer treatment, however, these patients who undergo tumor resection often relapse and develop metastases. There is an urgent need to develop molecular biomarkers capable of identifying high-risk melanoma and melanoma treatment. Previous studies have reported that KPNB1 is dysregulated in a variety of cancers, such as prostate cancer [10], colorectal cancer [11], and lung adenocarcinoma [30]. In addition, KPNB1 downregulation increased the cycle arrest of lung adenocarcinoma cells and induced apoptosis [30]. Prostate cancer cells with KPNB1 silencing exhibited low proliferation ability and tumor growth ability in vivo [10], which indicates the promoting effect of KPNB1 on the growth of cancer cells. Meanwhile, KPNB1 is also associated with the metastasis of cancer cells. Specifically, colorectal cancer cells with relatively low KPNB1 expression showed low migration and invasion capabilities [11]. Similar to previous studies, in vivo and in vitro experiments demonstrate that KPNB1 overexpression is beneficial to the survival and metastasis of melanoma cells. Our observations suggest that the development of drugs targeting KPNB1 as well as genetic tools may provide direction for the treatment of melanoma.

Malignant melanoma is stubborn to most existing chemotherapy and often develops resistance. Cisplatin is a well-known chemotherapy drug that has been used to treat many human cancers, including melanoma [31]. DDP, which is effective in many types of cancer, interferes with DNA repair mechanisms by cross-linking with purine bases on DNA, leading to DNA damage and further apoptosis [32]. However, DDP resistance is a phenomenon that occurs in many types of cancer, including melanoma [33, 34]. Therefore, exploring the molecular mechanisms and potential molecular targets that affect the drug resistance of cancer cells is of great significance for the treatment of cancer. Previous studies showed that knockdown of KPNB1 enhanced the sensitivity of cervical cancer cells to DDP [35]. Similarly, the present study suggested that melanoma cells with lower KPNB1 expression showed more apoptosis upon DDP exposure, which indicates that overexpression of KPNB1 in melanoma may contribute to the generation of DDP resistance. In summary, our results also suggest that KPNB1 may be a potential molecular target for melanoma therapy.

G3BP1 has been shown to function differently in different cancers [13, 19]. Herein, we demonstrated that G3BP1 plays a tumor-promoting role in melanoma progression. Interestingly, we found that KPNB1 only affected the protein expression of G3BP1 but not the mRNA level, and KPNB1 downregulation increased the ubiquitination of G3BP1 in melanoma cells, which is similar to previous [20]. Those observations suggest that KPNB1 may up-regulate G3BP1 expression by reducing its ubiquitination. As a nuclear transporter, KPNB1 downregulation may result in the imbalance of protein homeostasis in the cytoplasm and nucleus, leading to protein overload in the cytoplasm. Proteins that regulate protein stability accumulate in the cytoplasm, and the ubiquitin-proteasome pathway is activated in the cytoplasm. Previous studies showed that heat shock protein 70, an important regulator of protein degradation, accumulated in the cytoplasm in KPNB1-silenced glioblastoma cells [20, 36]. Therefore, in this study, the regulation of KPNB1 knockdown on the ubiquitination of G3BP1 may also be realized through the recruitment of ubiquitination-related proteins in the cytoplasm. Additionally, we also found that KPNB1 and G3BP1 were co-localized and interacted at the protein level. So far, although there is no evidence that KPNB1 can combine with downstream proteins to directly affect its ubiquitination level, the possibility of binding with G3BP1 to affect its ubiquitination is not excluded, which requires further studies in the future. We further demonstrated that G3BP1 mainly existed in the cytoplasm. KPNB1, as a nuclear transporter, did not affect the nuclear transport of G3BP1, indicating that KPNB1 is selective for downstream cargo transport. Moreover, we also demonstrate that KPNB1 binding to G3BP1 does not regulate its nuclear translocation.

Functional analysis indicated that G3BP1 knockdown reversed the effect of KPNB1 on the malignant phenotype of cells, indicating that the regulation of KPNB1 on the development of melanoma may be achieved through G3BP1. However, in addition to G3BP1, KPNB1 can also play a role in cancer by regulating the nuclear transport of substrates, such as E2F transcription factor 1 (E2F1) [7] and p65 [37], which play a promoting role in melanoma [38, 39]. Besides, KPNB1 could also directly interact with β-catenin to promote cell proliferation in human glioma cells [40]. These findings suggest that KPNB1 may be involved in the development of melanoma through multiple pathways, one of which may be the KPNB1/G3BP1 axis. In addition, previous studies have reported the potential molecular mechanism of G3BP1’s involvement in cancer development, and G3BP1 is related to multiple pathways, such as the p53 [17], the PI3K/AKT [41], and the STAT3 pathways [42], which was confirmed in this study. Those findings demonstrate that G3BP1 may be involved in the development of cancer through a variety of pathways, and also confirm the important role of G3BP1 in the development of melanoma.

In summary, this study mainly reports that KPNB1 may promote melanoma progression by stabilizing the G3BP1 protein. KPNB1-G3BP1 axis represents a potential therapeutic targetable node for melanoma.

## Supplementary information

Supplementary Figure 1

Supplementary Figure 2

Supplementary Figure 3

supplementary figure legends

Supplementary Tables

## Data Availability

The data that support the findings of this study are available on request from the corresponding author.
