# Supplementary figures and images for: Tumor-promoting properties of karyopherin β1 in melanoma by stabilizing Ras-GTPase-activating protein SH3 domain-binding protein 1
Source: Cancer Gene Ther. 2022 Jul 28;29(12):1939–50. doi: 10.1038/s41417-022-00508-8 (PMC9750864; doi:10.1038/s41417-022-00508-8)

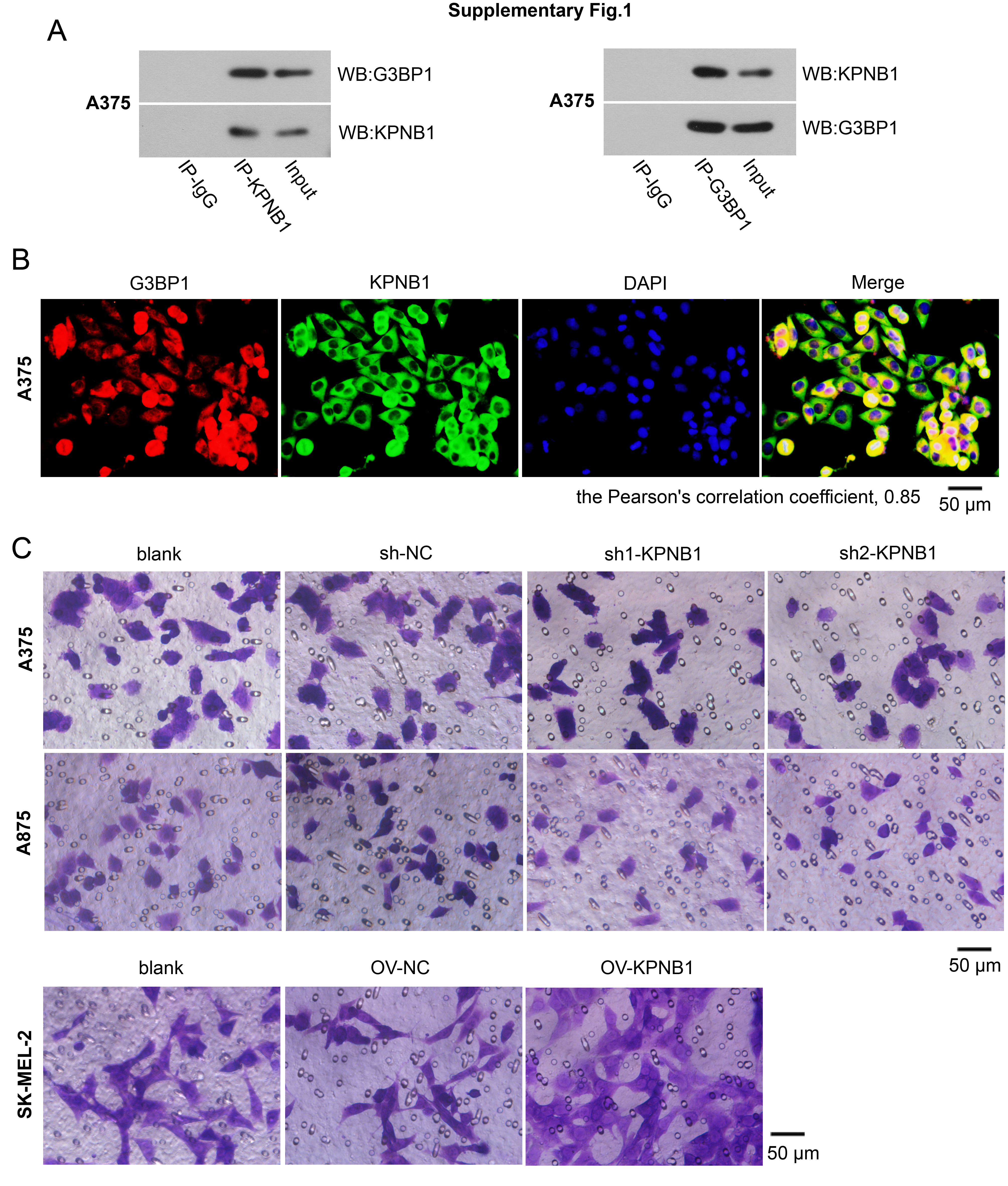

Supplement: Supplementary file 1 — Supplementary Figure 1 [file 41417_2022_508_MOESM1_ESM.tif]

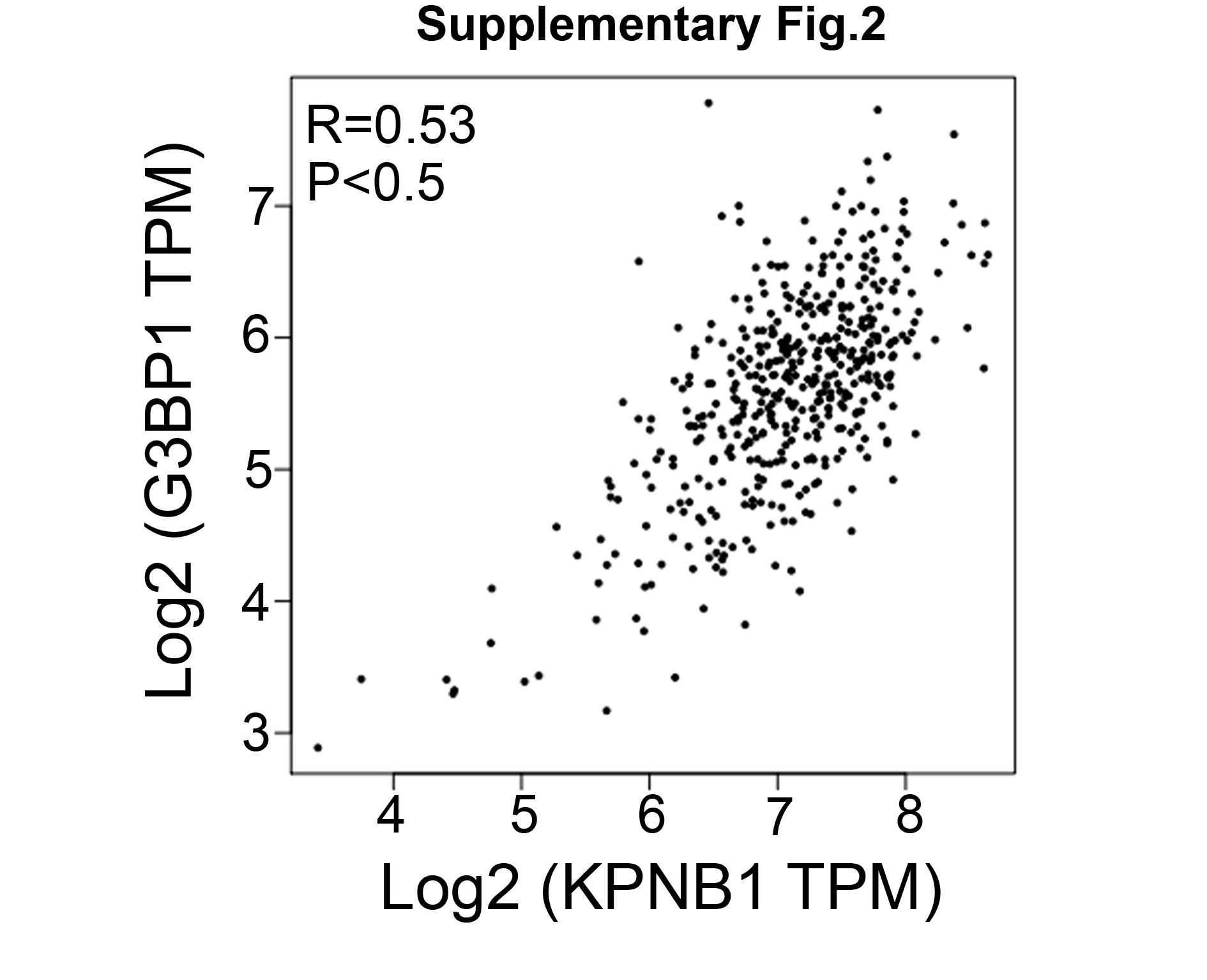

Supplement: Supplementary file 2 — Supplementary Figure 2 [file 41417_2022_508_MOESM2_ESM.tif]

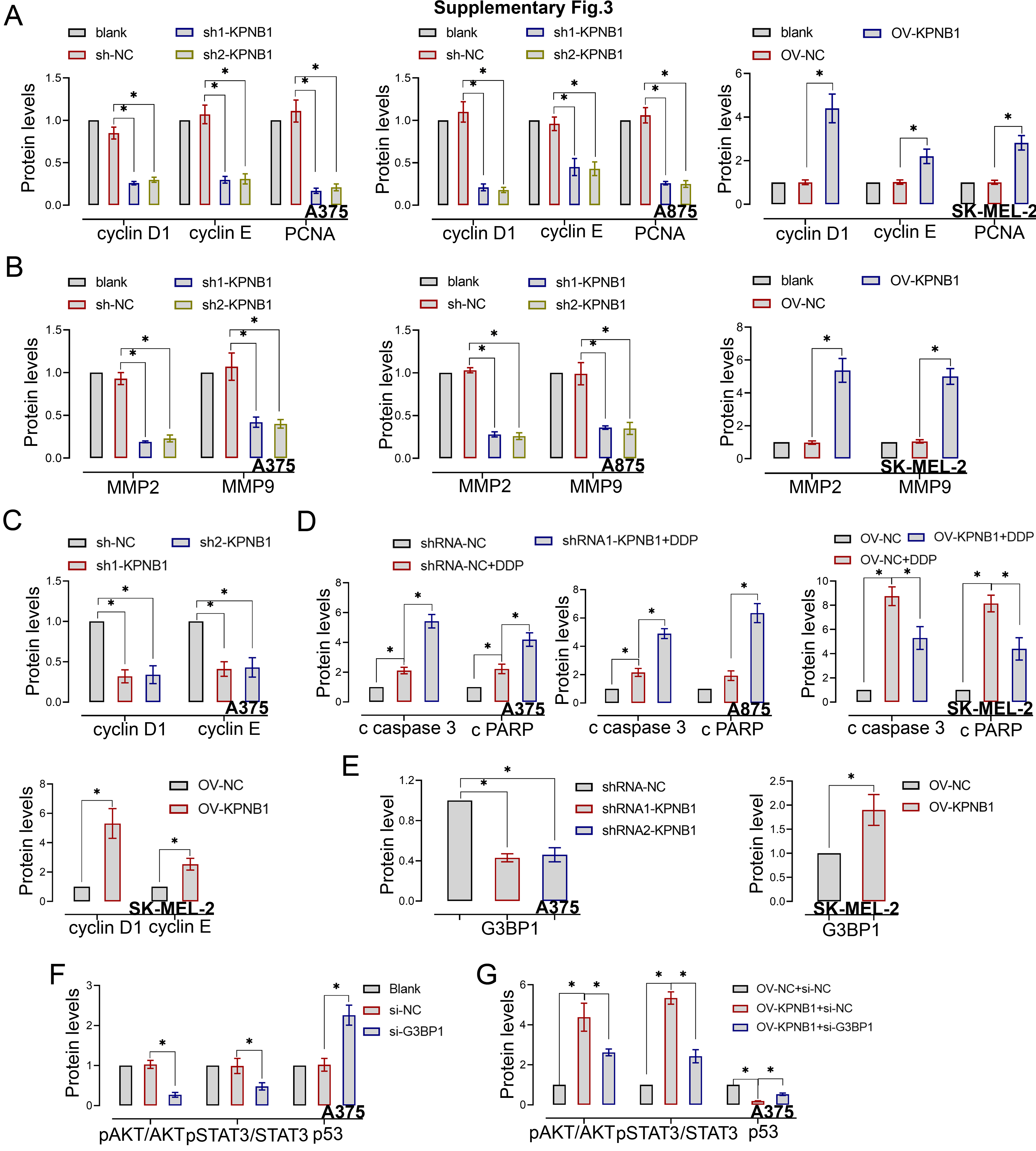

Supplement: Supplementary file 3 — Supplementary Figure 3 [file 41417_2022_508_MOESM3_ESM.tif]
