# Supplementary material for: Tumor-promoting properties of karyopherin β1 in melanoma by stabilizing Ras-GTPase-activating protein SH3 domain-binding protein 1
Source: Cancer Gene Ther. 2022 Jul 28;29(12):1939–50. doi: 10.1038/s41417-022-00508-8 (PMC9750864; doi:10.1038/s41417-022-00508-8)
Supplement: Supplementary file 4 — supplementary figure legends [file 41417_2022_508_MOESM4_ESM.docx]

**Supplementary Fig.1 (A)** Co-IP of KPNB1 with G3BP1 in A375 cells. **(B)** Immunofluorescence double staining of KPNB1 and G3BP1 in A375 cells. **(C)** Invasive cell numbers of melanoma cells with KPNB1 overexpression or silencing were assessed by Transwell assay. Scale Bar = 50 μm.

**Supplementary Fig.2** The correlation between KPNB1 and G3BP1 at the transcriptional level was analyzed by using the GEPIA website (<http://gepia.cancer-pku.cn/>).

**Supplementary Fig.3 (A-G)** Quantitative analysis of western blot results was performed. Data were shown as the mean ± SD. *P < 0.05 was determined by One-way ANOVA combined with Tukey's post hoc test or unpaired *t*-test.
