# Supplementary material for: Tumor-promoting properties of karyopherin β1 in melanoma by stabilizing Ras-GTPase-activating protein SH3 domain-binding protein 1
Source: Cancer Gene Ther. 2022 Jul 28;29(12):1939–50. doi: 10.1038/s41417-022-00508-8 (PMC9750864; doi:10.1038/s41417-022-00508-8)
Supplement: Supplementary file 5 — Supplementary Tables [file 41417_2022_508_MOESM5_ESM.docx]

Supplementary Table 1 Primer sequences used for qPCR.

| Genes | Sequences (5’-3’) |
| --- | --- |
| G3BP1 | Forward: TTTTCTTGGGCATCTGT |
|  | Reverse: CGTTGTTCTCGCACTCT |
| KPNB1 | Forward: GCTATGCCCACCCTAAT |
|  | Reverse: CAGCCAGACTGGAGAAA |
| β-actin | Forward: GGCACCCAGCACAATGAA |
|  | Reverse: TAGAAGCATTTGCGGTGG |

Supplementary Table 2 Primary antibodies used for Western blot.

| antibody | Manufacturer | Host and Clonality | Dilution |
| --- | --- | --- | --- |
| KPNB1 | Abcam (No. ab2811) | Mouse, mAb | 1:5000 |
| G3BP1 | Proteintech (No.13057-2-AP) | Rabbit, pAb | 1:5000 |
| Cyclin D1 | ABclonal (No. A19038) | Rabbit, mAb | 1:1000 |
| cyclin E | Affinity (No. AF0144) | Rabbit, pAb | 1:500 |
| MMP2 | Proteintech (No. 10373-2-AP) | Rabbit, pAb | 1:500 |
| MMP9 | Proteintech (No. 10375-2-AP) | Rabbit, pAb | 1:1000 |
| Cleaved caspase 3 | Affinity (No. AF7022) | Rabbit, pAb | 1:1000 |
| Cleaved PARP | CST (No. #5625) | Rabbit, mAb | 1:1000 |
| p-AKT | ABclonal (No. AP0637) | Rabbit, mAb | 1:500 |
| AKT | ABclonal (No. A17909) | Rabbit, mAb | 1:1000 |
| p-STAT3 | Affinity (No. AF3293) | Rabbit, pAb | 1:500 |
| STAT3 | Affinity (No. AF6293) | Rabbit, pAb | 1:1000 |
| p53 | Proteintech (No. 10442-1-AP) | Rabbit, pAb | 1:1000 |
| Histone H3 | Proteintech (No. 17168-1-AP) | Rabbit, pAb | 1:500 |
| β-actin | Proteintech (No. 60008-1-Ig) | Mouse, mAb | 1:2000 |

Supplementary Table 3 Primary antibodies used for CO-IP.

| antibody | Manufacturer | Host and Clonality | Dilution |
| --- | --- | --- | --- |
| KPNB1 (IP) | abcam (No. ab2811) | Mouse, mAb |  |
| KPNB1 (WB) | abclonal (No. A8610) | Rabbit, pAb | 1:500 |
| G3BP1 (IP) | proteintech (No. 13057-2-AP) | Rabbit, pAb |  |
| G3BP1 (WB) | proteintech (No. 66486-1-Ig) | Mouse, mAb | 1:10000 |
| Ubi (WB) | abcam (No. Ab7254) | Mouse, mAb | 1:10000 |
